# Systems Pharmacology-Based Strategy to Investigate Pharmacological Mechanisms of Radix Puerariae for Treatment of Hypertension

**DOI:** 10.3389/fphar.2020.00345

**Published:** 2020-03-24

**Authors:** Wenting Wu, Songhong Yang, Peng Liu, Li Yin, Qianfeng Gong, Weifeng Zhu

**Affiliations:** School of Pharmacy, Jiangxi University of Traditional Chinese Medicine, Nanchang, China

**Keywords:** Radix puerariae, hypertension, bioactive ingredients, mechanism of action, systems pharmacology

## Abstract

Hypertension is a clinical cardiovascular syndrome characterized by elevated systemic arterial pressure with or without multiple cardiovascular risk factors. Radix Pueraria (RP) has the effects of anti-myocardial ischemia, anti-arrhythmia, vasodilatation, blood pressure reduction, anti-inflammation, and attenuating insulin resistance. Although RP can be effective for the treatment of hypertension, its active compounds, drug targets, and exact molecular mechanism are still unclear. In this study, systems pharmacology was used to analyze the active compounds, drug target genes, and key pathways of RP in the treatment of hypertension. Thirteen active compounds and related information on RP were obtained from the TCMSP database, and 140 overlapping genes related to hypertension and drugs were obtained from the GeneCards and OMIM databases. A PPI network and a traditional Chinese medicine (TCM) comprehensive network (Drug-Compounds-Genes-Disease network) were constructed, and 2,246 GO terms and 157 pathways were obtained by GO enrichment analysis and KEGG pathway enrichment analysis. Some important active compounds and targets were evaluated by *in vitro* experiments. This study shows that RP probably acts by influencing the proliferation module, apoptosis module, inflammation module, and others when treating hypertension. This study provides novel insights for researchers to systematically explore the mechanism of action of TCM.

## Introduction

Hypertension is a clinical cardiovascular syndrome characterized by elevated systemic arterial pressure with or without multiple cardiovascular risk factors. It is also an important cause of and risk factor for a variety of cardiovascular and cerebrovascular diseases, affecting the structure and function of the heart, brain, kidney, and other important organs, eventually leading to organ failure (National Clinical Guideline Centre (UK), 2011; Moran et al., 2015).

Traditional Chinese medicine (TCM) has been used in clinical treatment in China for more than 2,500 years (Robinson, 2011). Compared with western medicine, TCM has different antihypertensive targets (Zhao et al., 2010). Traditional Chinese medicine has accumulated much experience in the treatment of hypertension, obesity hypertension, refractory hypertension, and other aspects (Xu and Chen, 2008; Wang and Xiong, 2012; Xiong et al., 2013). In TCM theory, hypertension belongs to the categories of “headache”, “vertigo”, and “true pain”. The main etiology of hypertension can be divided into emotional disorders, eating disorders, long-term illness, labor injury, congenital endowment, and so on. The main pathological links are wind, fire, phlegm, blood stasis, and deficiency, which are closely related to liver, spleen, kidney, and other viscera. The pathogenesis is based on deficiency of liver and kidney, Yin deficiency of liver and kidney, hyperactivity of liver Yang, and turbid sputum. Pathogenesis can be divided into liver-yang hyperactivity, phlegm-dampness, blood stasis, liver-kidney Yin deficiency, kidney-yang deficiency. Radix Pueraria (RP) not only has the effects of anti-myocardial ischemia, anti-arrhythmia, vasodilatation, blood pressure reduction, anti-inflammation, and attenuating insulin resistance (Zhang et al., 2010; Hwang et al., 2011; Yuan et al., 2014; Zhou et al., 2014; Wang et al., 2016; Yuan et al., 2016), but also can be used to treat hypertension. Especially Puerarin, a major compound in RP, can attenuate angiotensin II–induced cardiac hypertrophy by inhibiting the activation of the redox-sensitive ERK1/2, p38, and the NF-κB signaling pathways (Chen G. et al., 2014).

Although RP can be effective for the treatment of hypertension, its active compounds, drug targets, and exact molecular mechanism are still unclear. Each formula consists of a variety of Chinese medicines, each containing dozens to hundreds of compounds. Due to a variety of compounds can play a role of synergistic treatment on multiple targets, so the analysis of the mechanism of the formula is a time consuming and resource intensive process. Fortunately, systems-pharmacology has become a recent academic field, which incorporates a variety of disciplines and technologies, including biological chemistry, physiology, genetics, and computer science system pharmacology, provides a holistic approach to explore and understand the nature of the traditional medicine and its formula. Systems-pharmacology is able to identify compound-compound, compound-target, and target-disease interactions in computer models and understand the effects of herbs on biological networks based on system theory. At present, there are many methods based on systematic pharmacology to study the effect of traditional Chinese medicine on hypertension. The study shows that the potential mechanism of the therapeutic effect of traditional Chinese medicine on hypertension can be predicted through systematic pharmacology, so as to verify its therapeutic potential. According to systems pharmacology studies, San Cao Decoction can lower hypertension through regulating the pathway of PI3K-Akt-eNOS. And Oryeong-san formula may suppress hypertension by controlling the renin-angiotensin-aldosterone system (RAAS) (Kim et al., 2019; Ma et al., 2019). By using web-based approaches, systems pharmacology can systematically determine the effects and mechanisms of drugs used to treat complex diseases at the molecular, cellular, tissue, and biological levels. This research strategy for Danlu capsules, the Maxing Ganshi Decoction, the Wei Pi Xiao Decoction, Zuojinwan, and other capsules from traditional Chinese medicine has been widely adopted (Huang et al., 2017; Song et al., 2018; Yu et al., 2018; Yang et al., 2019).

**Figure 1:**
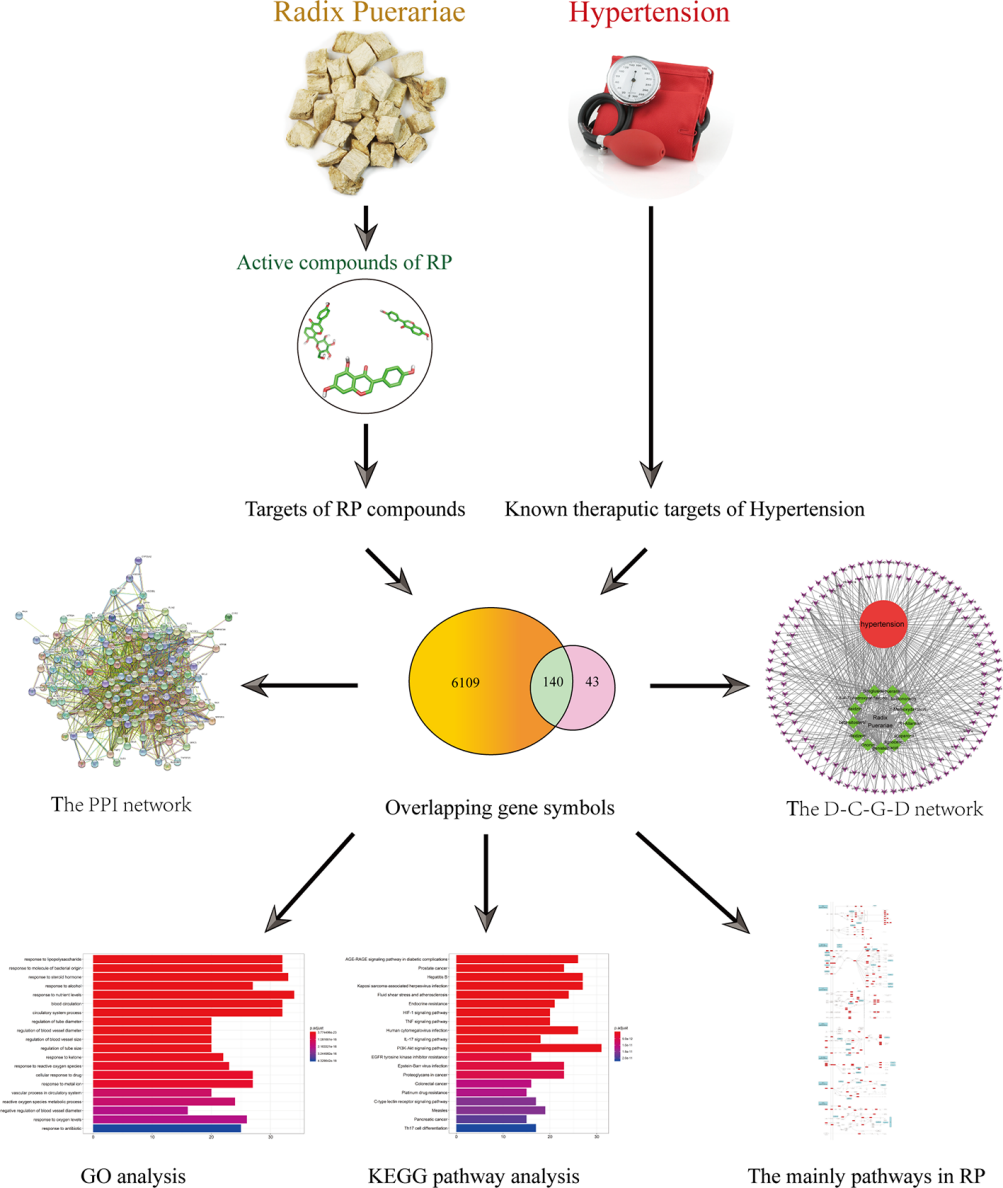
The flowchart of a systems pharmacology-based strategy to investigate the pharmacological mechanisms of Radix Puerariae for the treatment of hypertension.

In this study, systems pharmacology was used to analyze the active compounds, drug targets and key pathways of RP in the treatment of hypertension, as shown in **Figure 1**. This study attempts to provide a potential novel insight for researchers to systematically explore the mechanism of action of RP.

## Methods

### Screening of Active Compounds

All compounds contained in Radix Puerariae (RP) were searched from the Traditional Chinese Medicine Systems Pharmacology Database and Analysis Platform (TCMSP, http://tcmspw.com). TCMSP is a special systems pharmacology platform about Chinese traditional herbal medicines that includes the relationships between drugs, targets, and diseases. TCMSP has a wide range of 500 drugs and more than 12,000 compounds (Ru et al., 2014). It has a large number of chemical substances, targets, and drug target networks, as well as the corresponding drug target-disease network. It also includes the pharmacokinetic properties of natural compounds such as oral bioavailability, drug-likeness, intestinal epithelial permeability, etc.

Oral bioavailability (OB) refers to the percentage of unmodified drugs that enter the circulatory system after oral administration (Xu et al., 2012; Liu et al., 2013). OB is also an important indicator for objectively evaluating the internal quality of drugs (Alam et al., 2015). The higher the OB-value of a compound, the higher the likelihood that it can be used clinically. The drug-likeness (DL) is a vague concept that refers to the similarity between compounds and known drugs (Walters and Murcko, 2002; Tao et al., 2013). Compounds with drug-likeness properties are not drugs, but they have the possibility of becoming drugs. This class of compounds is called drug-like molecules or drug analogs. Therefore, the Tanimoto coefficient was used to evaluate the DL index of the molecule in RP. The formula is as follows:

(1) $$T(\alpha ,\beta )=\frac{\alpha \times \beta }{\alpha^{2}+\beta^{2}-\alpha \times \beta }$$

In this formula, α is the molecular property of the RP compound on the basis of Dragon software (http://www.talete.mi.it/products/dragon_description.htm). β means the average molecular property for all drugs in the DrugBank database (Mauri et al., 2006).

Because most compounds in Chinese medicine have poor pharmacological properties, they cannot effectively bind to specific cell protein targets. Thus, many researchers have recommended that the OB ≥ 30% and DL ≥ 0.18 molecules be considered to have better pharmacological effects and be selected as candidate compounds for further analysis (Chen G. et al., 2014; Song et al., 2018; Yu et al., 2018; Kim et al., 2019; Ma et al., 2019; Yang et al., 2019). This study also used this principle to screen compounds for further analyses.

### Identification of the Related Targets and Gene Symbols of the Compounds in RP

According to the principle of different compounds-target forecast, target prediction techniques and methods can be divided into four categories: Based on the predictions of a ligand (chemical similarity search and pharmacophore model); Based on the predictions of a receptor (molecular docking); Machine learning to predict (the molecules of a database must have a clear correspondence between with the targets, and the name of the target must be standardized); and Combination forecast. But different technologies have their limitations. For example, the defect of molecular docking method is that it is difficult to obtain target proteins with three-dimensional structure through experimental means or homologous modeling. However, the modeling process of machine learning prediction is blind and implicit, so the binding pattern between target proteins and compounds cannot be found directly. The data set used in the training model must contain accurate annotation information, which means that the small molecule must have a clear correspondence with the target and the target naming needs to be standardized, so the common compound database is not applicable. In addition, such methods may only focus on the receptor space of some protein families or are limited to the compound space of specific drugs, which is not ideal for the prediction of drugs and targets that are not in this space. Considering the limitations of the experimental conditions of molecular docking and machine learning prediction and combining that with previous experience, we finally chose the prediction method based on ligands for subsequent research (Yu et al., 2012; Wu et al., 2016; Wang S. et al., 2017).

All of the protein targets of the active compounds in RP were retrieved from TCMSP (http://lsp.nwu.edu.cn/tcmsp.php), removing redundant information and keeping only those that can interact directly with each of these compounds in RP as a presumption of targets. The target is then transformed using the UniProt knowledge database, and the selected species is *Homo sapiens* (Human). After deleting the redundant items, the data were merged to obtain the gene targets. UniProt is the most informative and well-resourced protein database. It is composed of data from three major databases: swiss-prot, TrEMBL, and pir-psd. The data come mainly from protein sequences obtained after the genome sequencing project was completed. It contains a wealth of information about the biological functions of proteins from the literature.

### The Acquisition of Gene Targets for Hypertension

This study collected gene targets for hypertension from two sources. The first one is the Gene Cards database (https://www.genecards.org/, version 4.9.0). Gene Cards is a searchable, comprehensive database that offers all comments and predicts human genetic information comprehensively in a user-friendly manner. It automatically integrates data from 150 web sources for the gene, including genome, transcriptome and proteome, genetics, and clinical and functional information (Rebhan et al., 1997; Safran et al., 2010). This study used the keyword “hypertension” to search this database. The second source is the Online Mendelian Inheritance in Man (OMIM) database. (http://www.omim.org/, updated on February 28, 2019) (Hamosh et al., 2005). It is a continuously updated database of human genes and genetic disorders. It focuses on genetic or inherited genetic diseases, including text information and relevant reference information, the sequence record, mapping, and other related databases. In this study, the keyword “hypertension” was searched by using the Gene map option in the advanced search field of this database. Eventually, we obtained genes for diseases associated with hypertension.

### Drug-Compounds-Genes-Disease (D-C-G-D) Network Construction

First, we intersected the obtained drug targets with the disease genes and obtained the Venn diagram of the intersected genes. Then, we built a network of complex information based on interactions between the drug (RP), compounds, genes and the disease (hypertension). Next, we used Cytoscape software (Shannon et al., 2003; Su et al., 2014) (version 3.7.1), which is a graphical display and network analysis and editing software, to perform a visual analysis of the “D-C-G-D network”.

### PPI Network Construction

Protein-protein interaction data were obtained from the String database (https://string-db.org/, version 11.0, updated on January 19, 2019) (Hsia et al., 2015). This is a database that searches for known proteins and predicts interactions between proteins, which is available for 5,090 species and contains interactions between 24,584,628 proteins and 3,123,056,667 proteins. The target is then transformed using the UniProt knowledge database. After deleting the redundant items, the data were merged to obtain the genes. At last, we searched for these genes in the database using the multiple proteins option, and at the same time, we set the organism to *Homo sapiens* (Human). A PPI network of RP active compounds-targets and hypertension-related targets was then constructed.

### Gene Ontology Enrichment Analysis

Gene Ontology (GO) is an international standard classification system for gene function. It is a standard of language vocabulary that can be used in all species. It can define and describe the functions of genes and proteins, and it can be regularly updated with new research. It can help researchers to focus on the biological functions of different genes from the perspective of gene function (Ashburner et al., 2000). This study used the bioconductor (R) software (http://bioconductor.org/, version 3.8, released on October 31, 2018) for analysis.

### KEGG Pathway Enrichment Analysis

The KEGG (Kyoto Encyclopedia of Genes and Genomes) database is a database systematically analyzing the metabolic pathways of gene products in cells and the functions of these gene products. The database is useful for studying genes and expressed information as a whole network. KEGG integrates data from the genome, chemical molecules and biochemical systems, including metabolic pathways, drugs, diseases, and gene sequences (Ogata et al., 1999). *In vivo*, different gene products coordinate with each other to perform biological functions, and pathway annotation analysis of differentially expressed genes helps further interpret gene functions. Overrepresentation of differentially expressed proteins on a given pathway shows the pathway enrichment analysis of differentially expressed proteins. This study also used the Bioconductor (R) software (http://bioconductor.org/, version 3.8, released on October 31, 2018) for analysis.

### Computational Validation of Compounds-Targets Interactions

We wished to ascertain the interaction between active compounds and their protein targets and explore their binding modes. Hence, we selected three active compounds and four targets, a total of six compounds-targets interactions for verification of molecular docking. We used GOLD v5.1 (a genetic algorithm-based docking program to dock protein-ligand complexes). We obtained the X-ray crystal structures of IL-6, IL-1β, IL-4, and Prostaglandin G/H synthase 2 (PTGS2) from the RCSB Protein Data Bank (PDB) (www.rcsb.org); the PDB entry code for these proteins is 1ALU, 2NVH, 2D48, and 5F19, respectively. During molecular docking, we adopted the GOLD Score fitness function.

### Experimental Validation

#### Reagents

Puerarin (purity ≥ 98%) and Daidzein (purity ≥ 98%) was purchased from Must Biotechnology (Chengdu, China). A stock solution of 100 mM puerarin or daidzein in dimethyl sulfoxide was prepared and stored at 4°C. Enzyme-linked immunosorbent assay (ELISA) kits for IL-6 and IL-4 were purchased from MultiSciences Biotech (Hangzhou, China).

#### Cell Culture

RAW264.7 cells were obtained from the cell bank of the Chinese Academy of Sciences (Shanghai, China). RAW264.7 cells were cultured in Dulbecco’s modified Eagle’s medium (Gibco, Billings, MT, USA) with 10% fetal bovine serum (Gibco). Cells were cultured at 37°C in an atmosphere of 5% CO2 for all experiments.

#### Assay to Measure Cell Viability

RAW264.7 cells in the logarithmic phase were seeded at 8 × 10^4^ cells/well in 96-well culture plates. After incubation for 24 h, Raw264.7 cells were exposed to puerarin or daidzein (0, 5, 25, 50, 75, or 100 μmol/L). After treatment for 24 h, 20 μl of Cell Counting Kit (CCK-8) assay solution (Biosharp, Hefei, China) were added to each well, and cells were incubated for 4 h at 37°C in an atmosphere of 5% CO2. The absorbance at 450 nm was measured by a microplate reader. Cell survival was calculated as: absorbance/absorbance of control ×100%.

#### Determination of Levels of IL-6 and IL-4 by ELISAs

RAW264.7 cells (1×10^6^ cells/well) were incubated with lipopolysaccharide (LPS; 1 μg/ml) for 24 h and then treated with daidzein (50, 75, or 100 μM) for 24 h. Supernatants were harvested and the level of IL-6 and IL-4 determined by ELISA kits (Biosharp).

#### mRNA Expressions of NF–κBIA, BCl2, and PTGS2 Treat With Puerarin by qRT-PCR

RAW264.7 cells (2×10^6^ cells/well) were incubated with lipopolysaccharide (LPS; 1 μg/ml) for 24 h and then treated with puerarin (50, 75, or 100 μM) for 24 h.

Total RNA was extracted with TRIzol^®^ Reagent (Thermo Scientific, Waltham, MA, USA), and reverse-transcribed with oligo-DT using HiScript™ Reverse Transcriptase (Vazyme, Beijing, China) according to manufacturer instructions. The primers used were synthesized by Genscript (Nanjing, China). The sequences were (forward and reverse, respectively) 5′-TTGGTCAGGTGAAGGGAGAC-3′ and 5′-GGATCACAGCCAGCTTTCAG-3′ for NF-κBIA; 5′-TTGCGTGAAGGCTTGAGATG-3′ and 5′-CTGGACAGGATGGAGGGTTT-3′ for BCL2; 5′-AAGCCTTCTCCAACCTCTCC-3′ and 5′-GCTGGGCAAAGAATGCAAAC-3′ for PTGS2; 5′-AACGGATTTGGCCGTATTGG-3′ and 5′-CATTCTCGGCCTTGACTGTG-3′ for the internal control glyceraldehyde 3-phosphate dehydrogenase (GAPDH).

qRT-PCR was done using SYBR™ Green Master Mix (Vazyme) in the QuantStudio 6 Flex system (Applied Biosystems, Foster City, CA, USA). The PCR cycling profile was: one cycle at 50°C for 2 min and 95°C for 10 min, 40 cycles at 95°C and 60°C for 30s. Fluorescence signals were detected using the QuantStudio 6 Flex system. Gene-expression data were normalized to that of the endogenous control GAPDH. The 2^−ΔΔCT^ method was the basis for relative expression of genes.

### Statistical Analyses

Data are the mean ± SD. The significance of results was determined based on one-way analysis of variance using Prism 8.0.1 (Graphpad, San Diego, CA, USA). p < 0.05 was considered significant. All experiments were repeated at least three times.

## Results

### Screening for Active Compounds

To identify the active compounds of RP, two classical ADME parameters, OB and DL, were used for screening. Some did not agree with the standard of screening compounds that are also likely to produce therapeutic effects on the human body. To study this issue more comprehensively, although they do not meet the screening criteria, the compounds were still retained as active compounds in this study. In the TCMSP database, there are 18 listed compounds of RP. Among them, only 4 compounds had an OB greater than 30.0% and a DL greater than 0.18. The DL of 16 compounds was greater than 0.18 and the OB of 6 compounds was greater than 30.0%. However, we found that puerarin, with an OB of 24.03%, is a well-known active compound in RP (Chen G. et al., 2014; Li et al., 2016; Tao et al., 2016; Li et al., 2017; Mocan et al., 2018). These studies showed that puerarin may be used as a new potential antihypertensive drug. It can improve the vascular insulin effect of patients with salt-sensitive hypertension and is beneficial against cardiovascular disease (Tan et al., 2017).

Similarly, the DL of allantoin is 0.03, which is far less than 0.18. However, studies have shown that allantoin has potential as a novel antihypertensive drug in the future (Chen M. et al., 2014). In addition, allantoin can also play a therapeutic role in diabetes through its antioxidant effect (Go et al., 2015). All these studies indicate that allantoin has strong drug activity. Scoparone is another compound with excellent pharmacological effects that has significant antihypertensive, anti-inflammatory, antioxidation, and anticoagulant effects (Huang et al., 1992; Cho et al., 2016; Xu et al., 2016; Jung et al., 2019). However, its DL is also well below 0.18. These examples demonstrate that although some compounds are not high for OB or DL, they do have high efficacy. Therefore, considering these factors, we decided to select all of the compounds related to RP in the TCMSP database as the candidate compounds. To sum up, a total of 18 compounds were selected as the candidate compounds of RP in this study. **Table 1** shows the thirteen active compounds from RP and their corresponding predicted OB, DL, and structure.

**Table 1:** Showing the thirteen active compounds from Radix Pueraria (RP) and their corresponding predicted oral bioavailability (OB), drug-likeliness (DL), and structure.

No.	Mol ID	Molecule Nmae	OB	DL	Structure
1	MOL000392	Formononetin	69.67	0.21	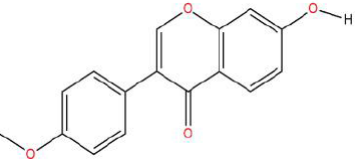
2	MOL000357	Sitogluside	20.63	0.62	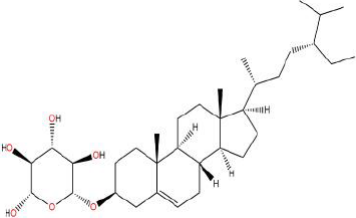
3	MOL000358	Beta-sitosterol	36.91	0.75	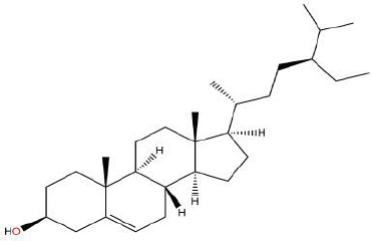
4	MOL000390	Daidzein	19.44	0.19	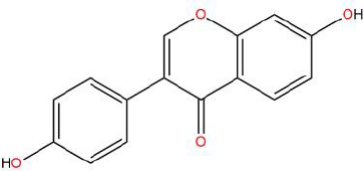
5	MOL000391	Ononin	11.52	0.78	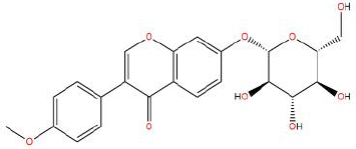
6	MOL000481	Genistein	17.93	0.21	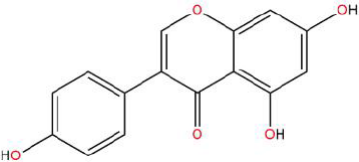
7	MOL000663	Lignoceric acid	14.90	0.33	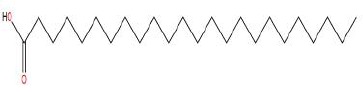
8	MOL001999	Scoparone	74.75	0.09	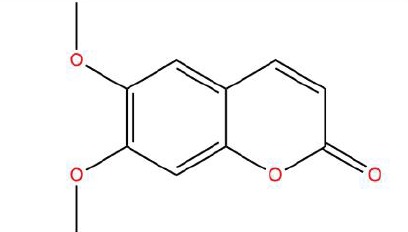
9	MOL002347	(R)-allantoin	96.90	0.03	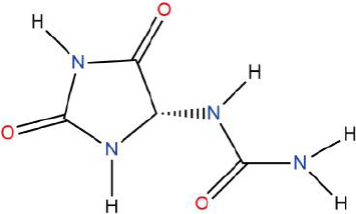
10	MOL002959	3’-Methoxydaidzein	48.57	0.24	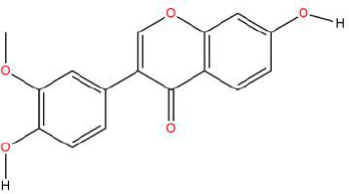
11	MOL012297	Puerarin	24.03	0.69	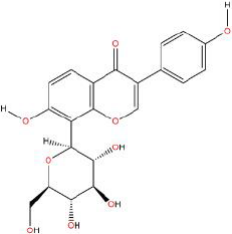
12	MOL004631	7,8,4’-Trihydroxyisoflavone	20.67	0.22	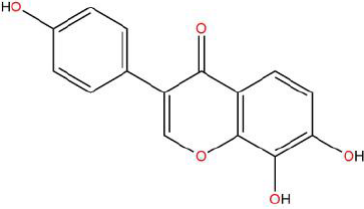
13	MOL009720	Daidzin	14.32	0.73	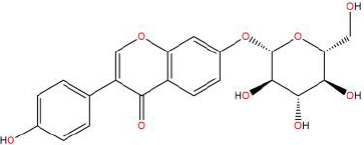

### Identification of the Related Targets and Genes of the Compounds in RP

After being collected from the TCMSP database and converted into the UniProt database, as well as deleting the redundant items, 13 active compounds in RP, and 183 known targets related to them were finally obtained. The details are described in Additional file: **Supplementary Table S1**.

### The Acquisition of Known Therapeutic Gene Targets for Hypertension

A total of 6,255 known therapeutic targets for hypertension were collected from the Gene Cards database. In addition, 11 known therapeutic targets for hypertension were obtained based on the OMIM database. After eliminating redundant targets, a total of 6,109 known therapeutic. Targets for hypertension were collected in this study. The details are described in Additional file: **Supplementary Table S2**.

### D-C-G-D Network Analysis

**Figure 2:**
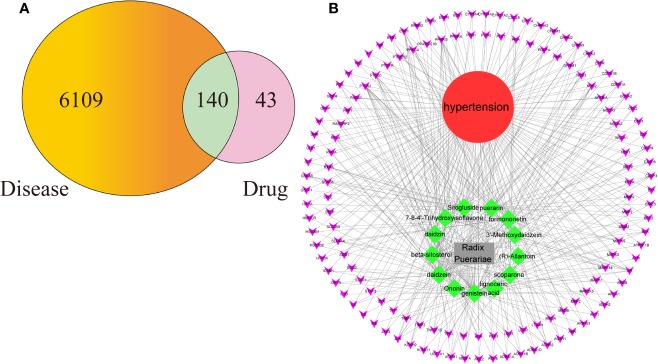
**(A)** The 140 overlapping genes between the disease and drug. **(B)** The Drug-Compounds-Genes-Disease (D-C-G-D) network.

**Figure 2A** shows that 6,109 disease genes and 183 drug genes have 140 overlaps. This means that these 140 genes may be the key to the treatment of hypertension by RP. The 140 overlapping genes are detailed in Additional file: **Supplementary Table S3**.

To clarify the potential mechanism of RP acting on hypertension, we used Cytoscape software to build the D-C-G-D network, as shown in **Figure 2B**. The gray node represents RP. The red node represents hypertension. The 13 green nodes represent the active compounds in RP. The 140 purple nodes represent the overlapping genes between the disease and drug. The edges mean that nodes can interact with each other.

We conducted further network analysis by evaluating centralization and heterogeneity. The network centralization and heterogeneity are 0.885 and 2.442 and are different from each other, showing that some nodes are more concentrated in the network than others. This means that the compound-target space has a tendency for certain compounds and targets. Therefore, the network includes some compounds with multiple targets, such as genistein (degree=66), daidzein (degree=49), puerarin (degree=45), formononetin (degree=26), beta-sitosterol (degree=25), and scoparone (degree=14). Additionally, it means that RP can act on multiple targets through the same active compound. For example, puerarin can have an effect on 45 targets, such as VEGFA, PTGS2, AR, PPARG, and RELA, when treating hypertension. Studies have shown that puerarin can achieve anti-inflammatory effects by activating the NF-κB signaling pathway and antioxidant effects by inhibiting the Nrf2 pathway (Li et al., 2018; Liu et al., 2019; Wang et al., 2019). Detailed information about the active compounds and genes are described in Additional file: **Supplementary Table S4**.

### PPI Network Analysis

**Figure 3:**
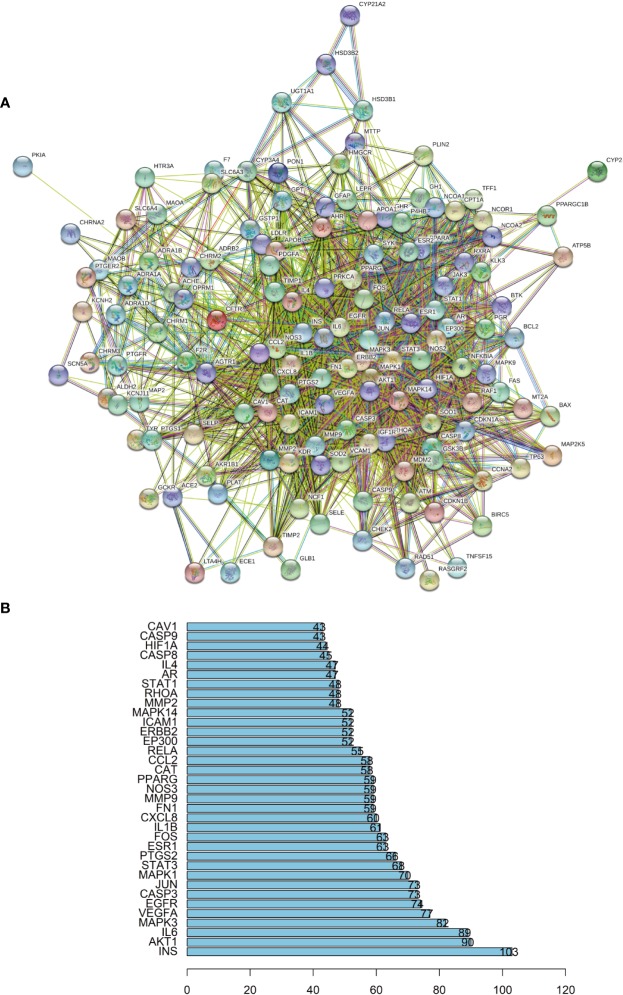
**(A)** The protein-protein interaction (PPI) network. **(B)** The bar plot of the protein-protein interaction (PPI) network. The X-axis represents the number of neighboring proteins of the target protein. The Y-axis represents the target proteins.

In this study, we constructed a PPI network of the 140 overlapping targets, which consisted of 140 nodes and 2,076 edges, as shown in **Figure 3A**. This means that the proteins have more interactions among themselves than would be expected for a random set of proteins of similar size drawn from the genome. Such an enrichment indicates that the proteins are at least partially biologically connected as a group. The light blue edges mean that the known interactions come from curated databases. The pink edges mean that the known interactions come from experimentally determined evidence. The green edges mean that the predicted interactions come from the gene neighborhood. The red edges mean that the predicted interactions come from the gene fusions. The dark blue edges mean that the predicted interactions come from gene co-occurrences. The yellow edges mean that the predicted interactions come from text mining. The black edges mean that the predicted interactions come from coexpression. The lavender edges mean that the others come from protein homology. The details of the PPI network are described in Additional file: **Supplementary Table S5**. We took the first 35 proteins in the PPI network for further analysis.

As seen in **Figure 3B**, proteins can be related to other proteins. This suggests that the therapeutic effect of RP on hypertension is likely to be achieved through multiple compounds, multiple targets, and multiple pathways.

### Gene Ontology Enrichment Analysis

**Figure 4:**
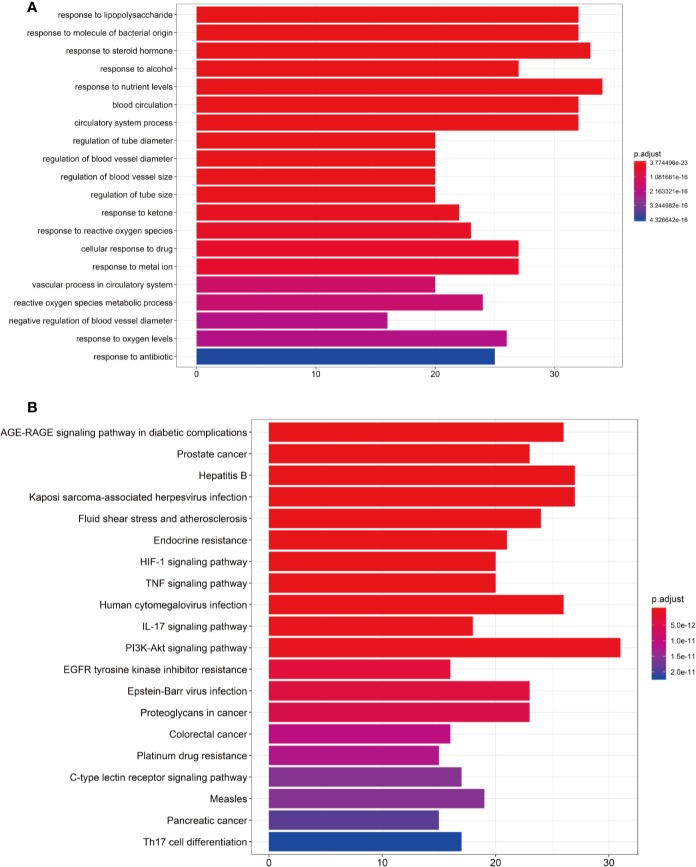
**(A)** Gene Ontology (GO) analysis of the 140 overlapping gene symbols associated with hypertension. The X-axis represents the significant enrichment counts of these terms, while the Y-axis represents the categories of “biological process” in the GO of the target genes (p-value < 0.01). **(B)** The Kyoto Encyclopedia of Genes and Genomes (KEGG) pathway enrichment analysis. The X-axis represents the target counts in each pathway, and the Y-axis represents the main pathways (p-value < 0.01).

To verify whether the 140 genes are related to hypertension, we conducted GO enrichment analysis to clarify the relevant biological processes, as shown in **Figure 4A**. The Y-axis represents the GO term. The X-axis indicates the number of genes enriched for the term. The redder the color, the smaller the value of p.adjust. This also means greater credibility and more importance. In contrast, the bluer the color, the greater the value of p.adjust. We used the first 20 terms from small to large according to the p-value for a brief demonstration. The details of the GO analysis are described in Additional file: **Supplementary Table S6**. The results indicated that numerous biological processes are involved in the treatment of hypertension, including response to lipopolysaccharide (GO:0032496), response to steroid hormone (GO:0048545), response to oxidative stress (GO:0006979), regulation of blood vessel diameter (GO:0097746), blood circulation (GO:0008015), regulation of blood vessel size (GO:0050880), negative regulation of blood vessel diameter (GO:0097756), and others.

### The KEGG Pathway Enrichment Analysis

The KEGG pathway enrichment analysis was performed by using the Bioconductor (R) software, as shown in **Figure 4B**. The Y-axis represents the pathway of the KEGG. The X-axis indicates the number of genes enriched in the pathway. The redder the color, the smaller the value of p.adjust, which also means more credibility and more importance. In contrast, the bluer the color, the greater the value of p.adjust. In this study, the 140 overlapping gene symbols were mapped to 157 pathways following KEGG pathway enrichment. We sorted the first 20 pathways from small to large according to the p-value for a brief demonstration. The details of the KEGG pathway enrichment analysis are described in Additional file: **Supplementary Table S7**.

**Figure 5:**
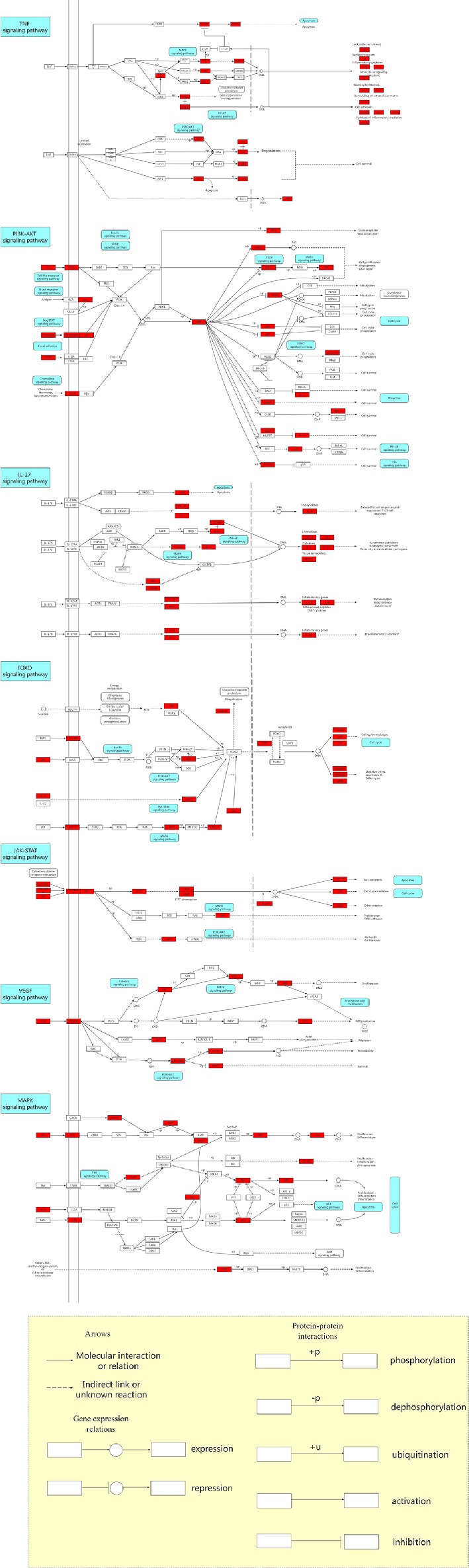
The Kyoto Encyclopedia of Genes and Genomes (KEGG) pathway map of anti-hypertension for Radix Pueraria (RP). The red nodes within the predicted signaling pathway represent the targets relevant with the corresponding pathway. The yellow area below refers to the explanation of the notations used in the pathway map.

According to the results, an integrated “RP pathway” has been constructed by the KEGG enrichment analysis, which can mainly be roughly divided into a proliferation module, apoptosis module, inflammation module, and others, as shown in **Figure 5**. In these pathways, the PI3K-Akt signaling pathway, TNF signaling pathway, IL-17 signaling pathway, FoxO signaling pathway, JAK-STAT signaling pathway, VEGF signaling pathway, MAPK signaling pathway, AMPK signaling pathway, NF-κB signaling pathway, calcium signaling pathway, mTOR signaling pathway, PPAR signaling pathway, cGMP-PKG signaling pathway, p53 signaling pathway, and Ras signaling pathway are considered the top priority. These signaling pathways can also interact with other pathways within the results.

In these modules, hypertension is thought to be associated with excessive endothelial cell proliferation (Hsueh and Anderson, 1992; Ueno et al., 2000; Landmesser et al., 2003). Studies have shown that hypertension can be treated by inhibiting the PI3K-Akt signaling pathway, increasing apoptosis and reducing cell proliferation (Intengan and Schiffrin, 2001; Wang et al., 2012; Sun et al., 2016). Activation of the PI3K-Akt signaling pathway and inhibition of apoptosis are involved in the pathogenesis of hypertension (Lin et al., 2013; Wu et al., 2013).

Chronic low-grade inflammation is one of the many putative mechanisms for hypertension, with elevated levels of inflammatory cytokines and activation of the immune system (Ruan and Gao, 2019). Some studies have suggested that the risk of hypertension is positively correlated with increased inflammation (Tomiyama et al., 2018; Jayedi et al., 2019). Moderate increases in blood pressure activate T cells, which in turn promote inflammation, which further increases blood pressure, leading to severe hypertension (Harrison et al., 2012). Studies have found that hypertension can be suppressed by reducing inflammation, and specific inflammatory pathways can improve blood pressure control (Mathieu et al., 2009; Chen et al., 2019).

Vascular endothelial growth factor receptor (VEGFR) can regulate the cardiovascular system (Olsson et al., 2006). The effects of VEGF-A on endothelial cells involves migration, proliferation, differentiation, and tube formation, leading to vascular germination and angiogenesis (Ferrara and Kerbel, 2005). There is evidence that the activation of the endothelin system is probably a reflection of the ECs activation status caused by the lack of VEGF, and it is a regulator of elevated blood pressure and, to a certain extent, may also be a regulator of renal injury (van den Meiracker and Danser, 2016). Inhibition of VEGF signaling leads to vascular dysfunction, kidney damage, and hypertension (Bhargava, 2009; Pandey et al., 2018).

### Computational Validation of Selected Ingredients-Targets Interactions

In general, the number and strength of a ligand bound to a receptor is determined largely by the inhibitory efficiency (Wang J. et al., 2017). Therefore, we explored the interactions and binding modes between the inflammatory factors IL-6, IL-4, IL-1β, and Prostaglandin G/H synthase 2 (PTGS2) with their active ingredients by molecular docking.

**Figure 6:**
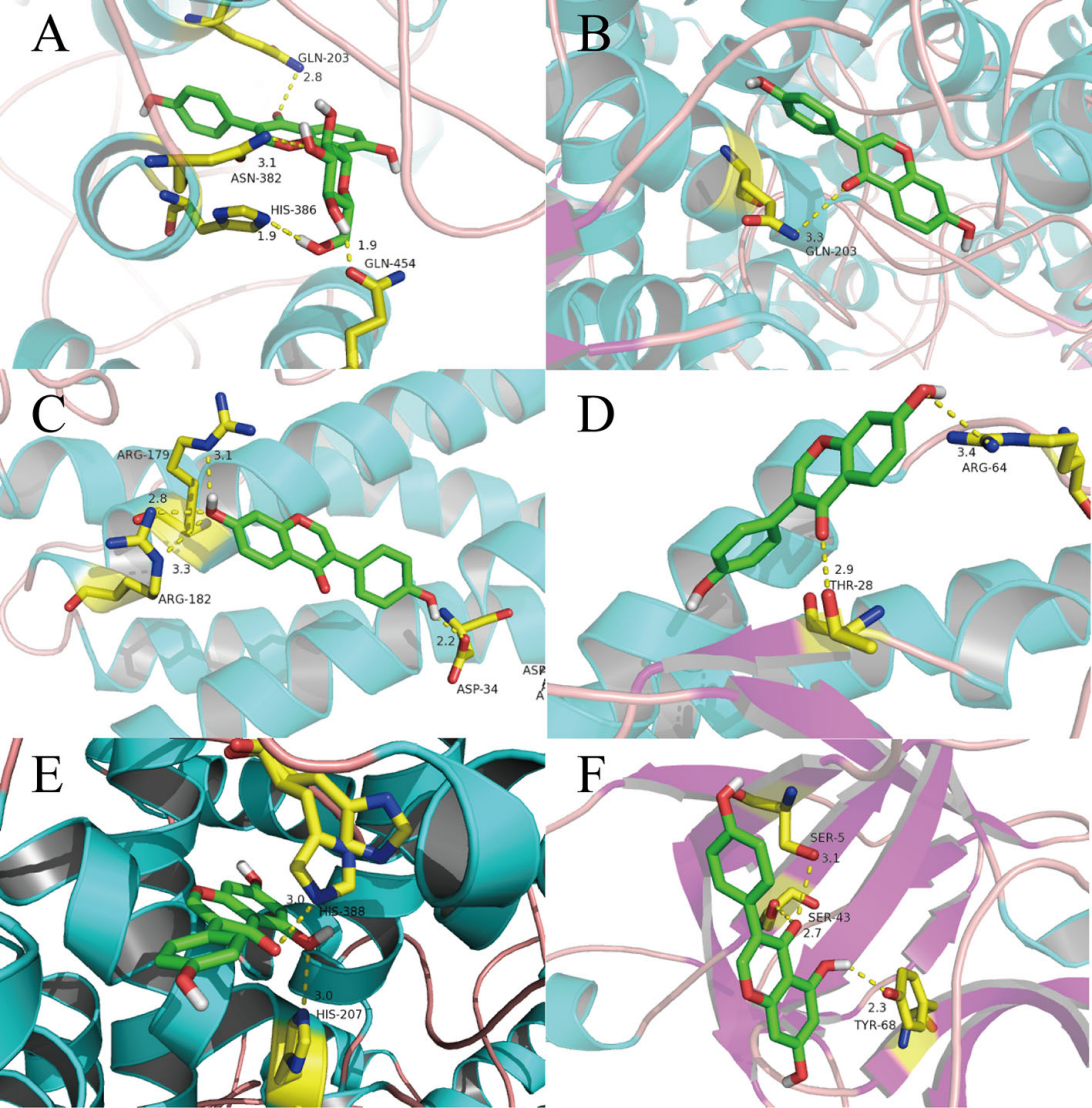
Binding explorations of selected Compounds-Targets interactions. **(A):** puerarin with PTGS2; **(B)** daidzein with PTGS2; **(C)** daidzein with IL-6; **(D)** daidzein with IL-4; **(E)** genistein with PTGS2;**(F)** genistein with IL-1β. Molecules are represented by a ball-and-stick model, the hydrogen bonds are represented by a dotted line, and the distance is in angstroms. Atoms C, O, and N are green, red, and blue, respectively.

Puerarin has special pharmacologic effects and high content in RP. Hence, we first conducted molecular docking of puerarin with PTGS2. We can see clearly from **Figure 6A** that the O atom in the C-O double bond in puerarin interacts with the N atom on GLN-203 (2.8 Å) through H-bond. An O atom in a -OH group of puerarin interacts with the N atom on ASN-382 (3.1 Å). The H atoms in the two other -OH groups in different positions in puerarin interacts with the N and O atoms on HIS-386 (1.9 Å) and GLN454 (1.9 Å), respectively.

In **Figure 6B**, the molecular-docking result of daidzein with PTGS2 shows that the O atom in the C-O double bond in daidzein can interact with the N atom on GLN-203 (3.3 Å).

In **Figure 6C**, we can see that daidzein with IL-6 has four connection points. The O atom in a -OH group and ARG-182 (2.8 Å) and ARG-182 (3.3 Å) interacts with each other between each N atom. The H atom in this -OH group can interact with ARG-179 (3.1 Å) between N atom. The H atom in the other -OH group can interact with the O atom on ASP-34(2.2 Å).

As shown in **Figure 6D**, the O atom in the C-O double bond in daidzein is formed the O atom on THR-28 (2.9 Å) in IL-4. A hydrogen-bond between the -OH group of the benzene ring and ARG-64 (3.4 Å) are formed.

Due to that genistein has the highest degree, the molecular docking of genistein with PTGS2 was performed, with results displayed in **Figure 6E**. From this figure, clearly two weak H-bonds are observed forming between the O atom and a -OH group of genistein and HIS-388 (3.0 Å) and HIS-207 (3.0 Å), respectively.

In **Figure 6F**, the H-bonding interactions between genistein and IL-1β, including SER-43…O (2.7 Å), SER-5…O (3.1 Å), and TYR-68…H (2.3 Å), make genistein and IL-1β complex remain a stable conformation.

The results of molecular docking were also consistent with our cell-experiment results, which demonstrated that puerarin and daidzein had a significant anti-inflammatory effect.

Based on these data, we can consider that the interaction between these active compounds and targets is the basis of their biologic activity. It also means that RP has multiple compounds and multiple targets.

### Experimental Validation *In Vitro*

#### CCK-8 Assay

**Figure 7:**
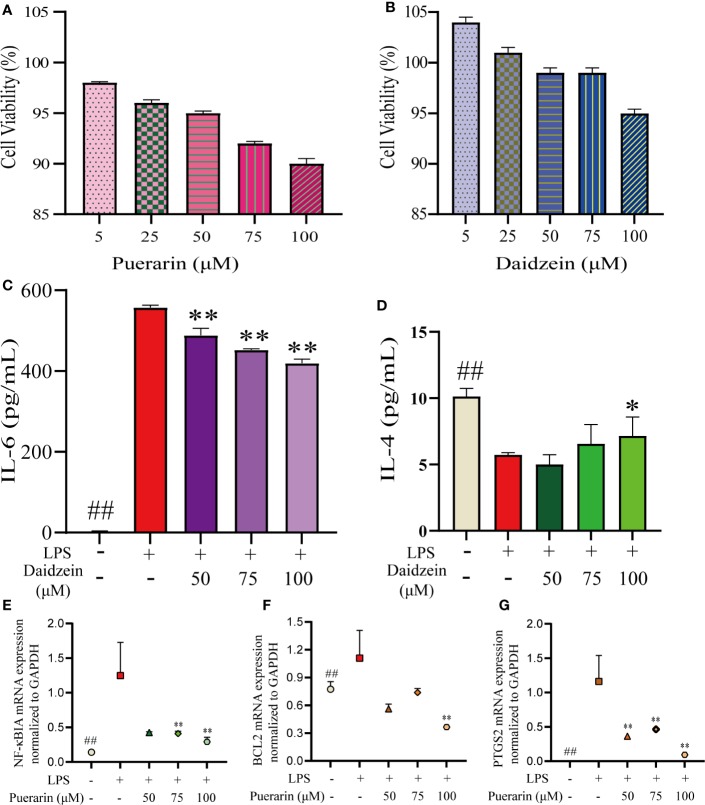
Results of puerarin and daidzein on RAW 264.7 cells. RAW264.7 cells were incubated with LPS (1 µg/mL) for 24 h and then treated with puerarin or daidzein (50, 75, or 100 µM) for 24 h. The effects of puerarin **(A)** or daidzein **(B)** on the viability of RAW264.7 cells using the CCK-8 assay. Production of IL-6 **(C)** and IL-4 **(D)** was determined by ELISAs. mRNA expression of NF-κBIA **(E)** and BCL2 **(F)** and PTGS2 **(G)** was determined by qRT-PCR. ##p < 0.01 versus blank control group. *p < 0.05 LPS-treated group. **p < 0.01 LPS-treated group.

First, we determined the effects of different doses of puerarin or daidzein on the viability of RAW264.7 cells using the CCK-8 assay (**Figures 7A, B**). Compared with the blank group, there was no significant decrease in the proliferation of RAW246.7 cells between puerarin or daidzein at the concentration of 5–100 μM. Therefore, three concentrations were selected (50, 75, 100 μmol/L) for subsequent experiments.

#### Validation of Targets

To further evaluate the results obtained in systematic pharmacologic analyses, puerarin, or daidzein was selected from RP to examine potential anti-inflammatory effects using LPS (1 μg/mL)-stimulated RAW264.7 cells. We undertook ELISAs for IL-6 and IL-4 and qRT-PCR for NF-κBIA and BCL2 and PTGS2 to confirm the predicted anti-inflammatory effects of the compounds. The production of IL-6 was suppressed obviously with the increase of the concentration of daidzein, whereas IL-4 was generated more (**Figures 7C, D**). Simultaneously, a significant decrease in NF-κBIA, BCL2, and PTGS2 mRNA expressions were observed (**Figures 7E–G**).

## Discussion

Hypertension is a clinical cardiovascular syndrome characterized by elevated systemic arterial pressure with or without multiple cardiovascular risk factors. It is also an important cause and risk factor for a variety of cardiovascular and cerebrovascular diseases, affecting the structure and function of the heart, brain, kidney, and other important organs, and eventually leading to organ failure (National Clinical Guideline Centre (UK), 2011; Moran et al., 2015). TCM has been used in clinical treatment in China for more than 2500 years (Robinson, 2011). Compared with western medicine, traditional Chinese medicine (TCM) has different antihypertensive targets (Zhao et al., 2010). Traditional Chinese medicine has accumulated much experience in the treatment of hypertension, obesity hypertension, refractory hypertension, and other aspects (Xu and Chen, 2008; Wang and Xiong, 2012; Xiong et al., 2013). However, its specific mechanism of pharmacological action has not been fully and comprehensively revealed (Wang et al., 2013a; Wang et al., 2013b; Xiong et al., 2014; Xiong, 2015). Therefore, it is imperative to explore the mechanism of RP in the treatment of hypertension by applying a systems pharmacology method combined with screening of active compounds, drug targets, and network and pathway analysis.

The results showed that there are 13 active compounds in RP influencing the 140 overlapping genes that play an important role in the process of treatment for hypertension. Puerarin is a well-known active compound in the treatment of hypertension (Zhou et al., 2014; Li et al., 2017). The therapeutic effect of puerarin on hypertension may be achieved by inhibiting the NF-κB pathway (Tan et al., 2017). Moreover, the key target of puerarin in reducing hypertension mechanism is believed to be NOS3, also called eNOS (Shi et al., 2019). The compound genistein from RP exhibited the highest number of interactions with various protein targets. Genistein can upregulate the expression of human endothelial nitric oxide synthase and lower blood pressure in spontaneously hypertensive rats and then treat hypertension (Xu et al., 2004; Cho et al., 2007; Si and Liu, 2008). It reduces hypertension through the PI3K/Akt/eNOS signaling pathway (Vera et al., 2007; Lin et al., 2012). In addition, daidzein has also been shown effective in treating hypertension (Furumoto et al., 2010).

On the other hand, the same target was found to be related to a variety of active compounds in RP, indicating that the active compounds in RP have a synergistic effect on the treatment of hypertension. PTGS2, namely, COX-2, is one of our predicted targets, which plays an important role in hypertension. It has been reported that selective cox-2 inhibition can treat hypertension through improving vascular diastole and reducing inflammation and oxidative stress (Chenevard et al., 2003; Hermann et al., 2003; Solomon et al., 2004; Martelli et al., 2013). It was predicted that PTGS2 can be linked to 12 active compounds in RP, which reflects that TCM works through multiple active compounds acting on a single target. Meanwhile, puerarin was speculated to be associated with 48 targets, such as PTGS2, VEGFA, CASP3, JAK3, SATA3, et al, in agreement with the fact that a single active compound could act on multiple targets for TCM. The PPI network also showed complex interactions among the 140 overlapping genes. Through the analysis of its compounds and targets, the network reveals that RP may treat hypertension through multiple pathways and cellular processes. According to the GO enrichment analysis and the KEGG pathway enrichment analysis, we speculate that RP may exert a therapeutic effect by impacting on the proliferation module, apoptosis module, inflammation module, and others.

In total, 2,246 GO terms (2,062 BP, 82 CC, 150 MF) and 157 pathways were obtained by GO enrichment analysis and KEGG pathway enrichment analysis. We thought that the PI3K-Akt signaling pathway, TNF signaling pathway, IL-17 signaling pathway, FoxO signaling pathway, JAK-STAT signaling pathway, VEGF signaling pathway, MAPK signaling pathway, AMPK signaling pathway, NF-kappa B signaling pathway, calcium signaling pathway, mTOR signaling pathway, PPAR signaling pathway, cGMP-PKG signaling pathway, p53 signaling pathway, and Ras signaling pathway might be principle pathways involved in the process of the treatment. The JAK-STAT cascade (GO:0007259) is associated with hypertension, and hypertension can be reduced by negative regulation of this cascade (Wold et al., 2002).

Systems pharmacology is an acceptable strategy to predict the therapeutic mechanism and can provide us research direction effectively. Considering the possible spurious associations between one data base with another, it would be significant to verify. Therefore, our investigation applied *in vitro* cellular experiments and molecular docking to prove the validity of the prediction to some extent through verifying the inflammation pathway. Our results demonstrated that puerarin and daidzein from RP may have a therapeutic effect against hypertension, and the inhibition of inflammation would probably one of their approaches to produce anti-hypertension effect, especially through the IL-17 and NF-κB signaling pathway. *In vitro* studies and molecular-docking results also provided additional information for the screened compounds with potential anti-inflammatory effects, and validated the reliability of this screening strategy based on systems pharmacology. Meanwhile, there are a series of reported studies on the effects of puerarin on anti-hypertension through *in vivo* models, such as cats and SHR, and all suggests that puerarin exerts its anti-hypertensive effect, which is coincident with our research (Wong et al., 2011; Wu et al., 2014). Savoia, C. et al. concluded in their article that inflammation participates in the development and pathogenesis of hypertension (Savoia and Schiffrin, 2006). In order to illustrate the mechanism more clearly, our future research will focus on in-vivo study and validation of the other pathways.

## Conclusions

Our research systematically investigates RP from a whole action mechanism perspective in the treatment of hypertension and provides a basis for multi-compound synergies in follow-up research and a new approach to explore traditional Chinese medicine. RP can achieve the effect of anti-hypertension through multiple compounds, multiple targets, and multiple approaches. A total of 13 active compounds and 140 overlapping genes between RP and hypertension were screened by systems pharmacology combined with screening of active compounds and target predictions. The PPI network, GO enrichment analysis and KEGG pathway analysis suggested that the pharmacological mechanism of RP in the treatment of hypertension may be related to its involvement in the proliferation module, apoptosis module, inflammation module, et al. In general, the systems pharmacological method developed in this study provides an alternative strategy for a comprehensive understanding of the mechanism of RP in the treatment of hypertension.

## Data Availability Statement

The data used to support the findings of this study are included within the article or within the **Supplementary Information File(s)**.

## Author Contributions

SY and WW conceived and designed the studies. PLcompleted in vitro experiments. LY participated in this work. All authors participated in drafting of the manuscript and revising it before final submission.

## Funding

This work was supported by The National Key Research and Development Plan (2017YFC1702900).

## Conflict of Interest

The authors declare that the research was conducted in the absence of any commercial or financial relationships that could be construed as a potential conflict of interest.

## Abbreviations

ADME, absorption, distribution, metabolism, and excretion; BCL2, Apoptosis regulator Bcl-2;DL, drug-likeness; GO, Gene Ontology; IL-1β, interleukin-1 beta; IL-4, interleukin-4; IL-6, interleukin-6; KEGG, Kyoto Encyclopedia of Genes and Genomes; LPS, lipopolysaccharide; NF-κBIA, scoparoneNF-kappa-B inhibitor alpha; OB, oral bioavailability; OMIM, Online Mendelian Inheritance in Man; PPI, protein-protein interaction; PTGS2, prostaglandin G/H synthase 2; RP, Radix Puerariae; STRING, Search Tool for the Retrieval of Interacting Genes/Proteins; TCM, traditional Chinese medicine; TCMSP, Traditional Chinese Medicine Systems Pharmacology Database.
